# Mapping the Gene Expression Spectrum of Mediator Subunits in Response to Viroid Infection in Plants

**DOI:** 10.3390/ijms21072498

**Published:** 2020-04-03

**Authors:** Vishnu Sukumari Nath, Ankita Shrestha, Praveen Awasthi, Ajay Kumar Mishra, Tomáš Kocábek, Jaroslav Matoušek, Andrej Sečnik, Jernej Jakše, Sebastjan Radišek, Vipin Hallan

**Affiliations:** 1 Department of Molecular Genetics, Biology Centre of the Czech Academy of Sciences, Institute of Plant Molecular Biology, Branišovská 31, 370 05 Ceske Budejovice, Czech Republic; sukumari.nath@umbr.cas.cz (V.S.N.); ankita.shrestha@umbr.cas.cz (A.S.); praveen.awasthi@umbr.cas.cz (P.A.); kocabek@umbr.cas.cz (T.K.); jmat@umbr.cas.cz (J.M.); 2 Department of Agronomy, Biotechnical Faculty, University of Ljubljana, Jamnikarjeva 101, SI-1000 Ljubljana, Slovenia; Andrej.Secnik@bf.uni-lj.si (A.S.); jernej.jakse@bf.uni-lj.si (J.J.); 3 Plant Protection Department, Slovenian Institute of Hop Research and Brewing, Cesta Žalskega Tabora 2, SI-3310 Žalec, Slovenia; sebastjan.radisek@ihps.si; 4 CSIR-Institute of Himalayan Bioresource Technology, Palampur 176061, India; hallan@ihbt.res.in

**Keywords:** differential expression, hop, mediator complex, *Nicotiana benthamiana*, *Nicotiana tabacum*, pathogen, quantitative reverse transcription PCR, viroid

## Abstract

The mediator (MED) represents a large, conserved, multi-subunit protein complex that regulates gene expression through interactions with RNA polymerase II and enhancer-bound transcription factors. Expanding research accomplishments suggest the predominant role of plant MED subunits in the regulation of various physiological and developmental processes, including the biotic stress response against bacterial and fungal pathogens. However, the involvement of MED subunits in virus/viroid pathogenesis remains elusive. In this study, we investigated for the first time the gene expression modulation of selected MED subunits in response to five viroid species (*Apple fruit crinkle viroid* (AFCVd), *Citrus bark cracking viroid* (CBCVd), *Hop latent viroid* (HLVd), *Hop stunt viroid* (HSVd), and *Potato spindle tuber viroid* (PSTVd)) in two model plant species (*Nicotiana tabacum* and *N. benthamiana*) and a commercially important hop (*Humulus lupulus*) cultivar. Our results showed a differential expression pattern of MED subunits in response to a viroid infection. The individual plant MED subunits displayed a differential and tailored expression pattern in response to different viroid species, suggesting that the MED expression is viroid- and plant species-dependent. The explicit evidence obtained from our results warrants further investigation into the association of the MED subunit with symptom development. Together, we provide a comprehensive portrait of MED subunit expression in response to viroid infection and a plausible involvement of MED subunits in fine-tuning transcriptional reprogramming in response to viroid infection, suggesting them as a potential candidate for rewiring the defense response network in plants against pathogens.

## 1. Introduction

Mediator (MED) is an evolutionarily conserved multi-subunit protein complex consisting of 25 to 34 subunits in eukaryotes, structurally organized into head, middle and tail modules and a dissociable cyclin-dependent kinase 8 (CDK8) module [1]. In the eukaryotic system, the MED serves as an integrative hub for RNA polymerase (RNAP) II-mediated transcription regulation through interaction with enhancer-linked general transcription factors (such as TFIIA, TFIIB, TFIID, TFIIE, TFIIF and TFIIH), facilitating the assembly of the pre-initiation complex (PIC) on gene promoters [2,3]. The tail module of the MED complex is recruited to enhancer or upstream activating sequence regions of genes by interactions with transcription factors (TFs) bound to these regions, and after an enhancer–promoter gene loop formation, the head and middle modules (thus forming the “core” mediator) interact with RNAPII and contribute to the recruitment and/or stabilization of PIC assembly, and phosphorylation of the RNAPII carboxy-terminal domain (CTD) by TFIIH, which causes the release of RNAPII from the promoters, thereby enabling the transition from transcriptional initiation to productive elongation [4,5,6]. The four subunit CDK8 module exchange is a reversible association with the MED complex, and in the association the CDK8 module intervenes in CTD-dependent RNAPII binding and suppresses transcription [7]. Various biochemical techniques and biophysical structural analyses have revealed 21 conserved subunits and six additional plant-specific subunits in *Arabidopsis thaliana*, whose positions in the complex are unassigned [8]. The physiological and cellular functions of several plant MED subunits have been deciphered by different forward/reverse genetic approaches and specific phenotypes of viable individual subunit mutants [9]. In *Arabidopsis*, for instance, the MED25 subunit is involved in the regulation of diverse physiological processes such as flowering, organ development, hormone signaling pathways and stress response [10,11,12]. The MED8, MED17, MED18 and MED20a subunits are involved in the production of non-coding RNA [13]. It has been shown that the MED8 subunit plays a role in normal pollen tube growth, whereas in tomatoes the MED18 subunit is required for pollen viability and the development of anthers [14]. The MED5a/MED5b subunits are responsible for maintaining the homeostasis of secondary metabolism [15]. Nevertheless, since the discovery of the MED complex, several studies have investigated its fundamental role in plant immunity.

The accumulating evidence suggests that the MED subunits (MED8, MED15, MED16, MED18, MED21, MED25 and CDK8) positively regulate resistance against leaf-infecting biotrophic bacteria or necrotrophic fungi via physical interaction with TFs and their integration into the phytohormone signaling network [10,16,17,18,19]. For example, the MED16 and MED25 subunits have been found to interact strongly with WRKY33 and MYC2 TF, respectively, which mediate jasmonic acid (JA)- and JA/ethylene (ET)-dependent defense responses [18,20]. Together, these studies provided a mechanistic understanding of the involvement of MED subunits in the triggering of defense signaling pathways in bacterial, biotrophic and necrotrophic fungal pathogenesis, but the role of MED subunits in the response of viruses/viroids will be key to expanding our understanding of plant immunity.

Viroids are unencapsidated, covalently closed, non-coding circular RNA molecules consisting of 246 to 371 nucleotides, and are etiological agents of devastating diseases in both monocots and dicots, including herbaceous, ligneous, agronomic and ornamental plants [21]. Phylogenetic reconstructions and structural and biological properties classified the viroids into two main families, *Avsunviroidae* and *Pospiviroidae* [22]. The members of *Avsunviroidae* (type species: *Avocado sunblotch viroid*) exhibit ribozyme-like self-cleavage activity, and replicate and accumulate in the chloroplast or plastid via a symmetrical rolling-circle mechanism using host enzymes, whereas the members of *Pospiviroidae* (type species: *Potato spindle tuber viroid*) consist of a rod-like secondary structure, and replicate and accumulate in the nucleus via an asymmetric rolling-circle mechanism utilizing host RNAPII [23,24]. The molecular mechanism underlying viroid pathogenesis remains elusive, but it is generally believed that the interference of viroid-derived small RNAs (vd-sRNAs) with the plant’s RNA silencing machinery, the methylation of host genes and the direct interaction of the mature viroid RNA motifs with the host proteins are underlying mechanisms of viroid pathogenesis [21,25,26].

More recently, genome-wide analyses in different viroid-host interactions such as *Potato spindle tuber viroid* (PSTVd)-infected tomato [27], *Peach latent mosaic viroid* (PLMVd)-infected peach [28], *Citrus bark cracking viroid* (CBCVd), *Hop latent viroid* (HLVd) and *Hop stunt viroid* (HSVd)-infected hop [29,30,31] have shown the dynamic modulation of genes involved in protein, sugar metabolism, photosynthesis, physiology, phytohormone signaling pathways, plant defense responses and cell wall structure. Intriguingly, our recent genome-wide analyses of *Humulus lupulus* (hop), in response to HLVd and CBCVd single and mixed infections [30,31], revealed an explicit notion of differential modulation of multiple MED subunits. In such a scenario, it was encouraging and instrumental to gain comprehensive insights into the response of MED subunits in different viroid–host combinations. In this report, for the first time, we have investigated the response of MED subunits to a viroid infection using different viroid–host combinations, which we hope will lay down a foundation for further studies delineating the detailed function of the plant MED complex in viroid pathogenesis.

## 2. Results and Discussion

Viroids have always been bewildering exceptions to the rules that characterize infectious agents due to their lack of the functional open reading frame that is generally accepted for other plant pathogenic RNAs. Over the last three decades, research on viroids has unexpectedly brought about several surprises that have significantly changed the general overview of biological processes, including the immune system of plants. In this context, the potential involvement of the MED subunits in the regulatory network of viroid pathogenesis could offer a new and fascinating perspective on elucidating the complex interactions that lead to symptom formation. To the best of our knowledge, this study reports for the first time the effects of viroid infection on the gene expression of MED subunits in plants. In this study, we selected five viroid species as a model system to study 19 MED subunits’ transcript responses in three plant species.

**Figure 1:**
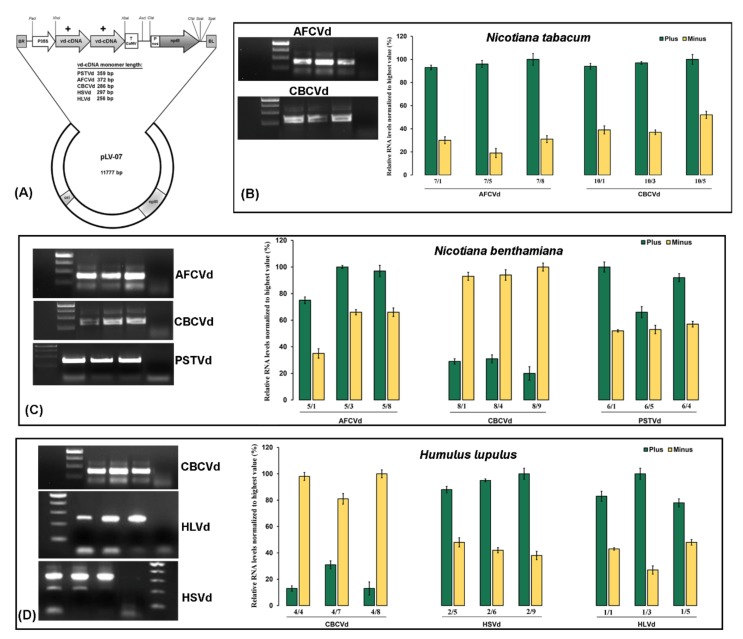
Schematic representation of dimeric infectious constructs, detection and quantification of viroids. (**A**) Schematic diagram of a plasmid containing the shown viroid (+) dimer created by cDNA cloning in SacI restriction site. The viroid (+) dimer was re-cloned from pPCR-Script to *Xho*I*–Xba*I sites of intermediary vector pLV-68. The final modified binary expression cassette harboring CaMV 35S promoter, viroid cDNA and CaMV terminator was cloned into *Pac*I and *Asc*I sites of the plasmid pLV-07. ori: origin of replication; kanR: kanamycin resistance gene; RB: left border of T-DNA; RB: right border of T-DNA; T CaMV: terminator from cauliflower mosaic virus; Pnos: nopalin synthase promoter; nptII: neomycin phosphotransferase II. RT-PCR-based detection and strand-specific real-time RT-qPCR quantification of viroids in single infected *Nicotiana tabacum* (**B**), *N*. *benthamiana* (**C**) and hop (**D**) plants. The gel picture shows three biological replicates of infected samples (with amplification) and a negative control (without amplification). The numbers under the bar indicate plant sample codes. All samples were normalized to the strand with a higher level (100%) and relative quantities were calculated using target-specific amplification efficiencies. Each column represents the mean ± SD of three technical replicates of single infected plants.

The viroid RNAs were detected by RT-PCR in the upper systemic leaves of all *N. benthamiana* plants agroinoculated with the PSTVd, CBCVd and AFCVd viroid transcripts, with moderate symptoms (reduced branching and early flowering) observed in these plants (Figure S1). In the case of tobacco inoculated with cDNA dimeric constructs of CBCVd and AFCVd on fully expanded three-week-old leaves, AFCVd RNA accumulation was detected in the upper systemic leaves, while CBCVd RNA accumulation was detected only in inoculated leaves by RT-PCR and ssRT-qPCR, indicating the absence of systemic trafficking across different cellular boundaries in an inoculated leaf due to the restriction of long-distance movement within the phloem. In the pre-dormancy period, hop plants individually infected with HSVd and CBCVd developed some typical symptoms such as the leaves curling downwards and mild yellowing, while plants infected with HLVd were asymptomatic. After dormancy, the most characteristic symptoms, such as bine cracking and stunted growth, were more pronounced in HSVd- and CBCVd-infected hop, whereas HLVd-infected plants remained asymptomatic (Figure S1). The high-fidelity RT-PCR product confirmed the individual CBCVd, HLVd and HSVd infection in hop samples (Figure 1). Nevertheless, ssRT-qPCR confirmed active viroid replication and the trend of an excess of multimeric plus or minus forms in infected samples (Figure 1). The gene expression response of MED subunits was evaluated using the three biological replicates and three technical replicates from the viroid-infected and mock-inoculated samples.

**Figure 2:**
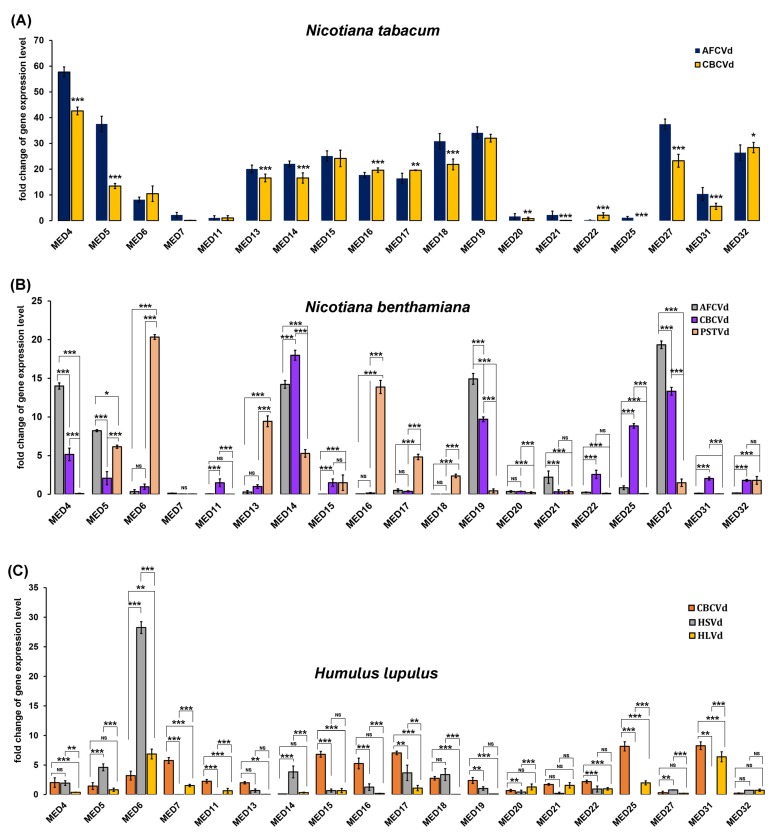
Reverse transcriptase quantitative real-time PCR (RT-qPCR)-based expression profiling of selected mediator subunits in response to the viroid infection in *N. tabacum* (**A**), *N*. *benthamiana* (**B**) and hop (**C**) plants. RT-qPCR analyses were normalized using *DRH1* as an internal control gene and the fold change in each gene in viroid infected/transformed plants was calculated with respect to mock-inoculated control plants by the (2^−ΔΔCt^) method. The data were obtained from three independent experiments; bars show ± SD. Comparison between groups was assessed by a two-way ANOVA followed by Tukey’s test, an asterisk denotes statistically significant differences (* *p* < 0.1, ** *p* < 0.05, and *** *p* < 0.01).

The RT-qPCR assay showed the varying expression patterns of the different MED subunits in the viroid-infected hop, tobacco and *N. benthamiana* (Figure 2), suggesting that viroids can trigger changes in the expression pattern of the MED subunits in plants. Nevertheless, AFCVd, CBCVd, HSVd, HLVd and PSTVd-infected hop, *N. tabacum* and *N. benthamiana* displayed the differential expression pattern of the same and different MED subunits (Figure 2), suggesting that the expression of MED subunits during viroid infection is dependent on the plant and viroid species. The growing body of knowledge suggested that MED subunits do not function in isolation, but rather are interdependent and often rely on each other for the regulation of vital biological processes, including biotic and abiotic stress [32,33]. In our study, several MED subunits displayed varied expression patterns depending on the plant and viroid species, underpinning their interdependent and coordinated regulation in response to viroid infection.

One of the essential roles of the MED complex is to transmit regulatory signals from TFs and promote mRNA biogenesis by recruiting the RNAP II to the promoter of protein-coding genes by facilitating the assembly of PIC [32,34,35]. The increasing evidence suggests that overexpression of the MED subunits’ genes elevates the expression of their immediate target genes [36]. The elevated and tailored expression of the MED subunits observed in our study, coupled with several previous reports about the massive modulation of genes involved in immune responses, primary and secondary metabolism and hormone signalling pathways [30,37,38,39], provided circumstantial support for the prominent role of the MED complex in the orchestrated transcriptional reprogramming of host genes in response to viroid infection.

More explicitly, several transcriptomic studies have shown that viroids extensively modulate the expression signatures of major phytohormone defense signaling pathways such as salicylic acid SA and JA [27,31]. Recent research has positioned the MED complex as the core regulator of phytohormone defense signaling [40]. In *A. thaliana*, MED subunits, namely MED14, MED15, MED16 and MED18, were found to be implicated in the regulation of the expression of defense genes during the immune response, especially in the SA- and JA-mediated pathways [41,42,43]. In the present study, we found consistent up-regulation of MED14, MED15 and MED16 (tobacco), and MED14 and MED19 (*N. benthamiana*) in response to both AFCVd and CBCVd infection, illustrating the plausible role of these MED subunits in triggering viroid-specific SA- and JA-mediated defense response pathways in tobacco and *N. benthamiana* plants, which warrants further investigation.

It has been reported that the MED subunits MED17, MED18 and MED20 regulate the production of non-coding RNAs [44]. The frameshift mutation in MED17, MED18 and MED20 subunits leads to the reduced accumulation of miRNAs, indicating their crucial role in miRNA biogenesis [44]. We have already shown that CBCVd infection can significantly alter the expression profile of miRNAs in hop [45]. The upregulation of MED17 and MED18 in CBCVd-infected hop suggested their plausible involvement in the modulation of miRNA expression in viroid pathogenesis. Recent work has extended the MED complex function from transcription to rRNA processing and ribosome biogenesis via interaction with RNAP I and III [46]. Correlated with the recent finding that defective ribosome biogenesis is directly related to viroid symptoms due to changes in rRNA processing, it is tempting to speculate that the MED complex could demarcate symptomatic and asymptomatic infection. However, a more detailed picture of the current state of knowledge on viroid-induced symptom development is solicited, which provides a paradigm for the investigation of specific protein–protein interfaces formed by the MED complex, disruption of which leads to symptom development.

To date, little is known about how the disruption of a particular MED subunit could affect the function of other subunits or the whole complex. Newly emerging reports have highlighted that, despite the critical nature of the MED complex, the disruption of some genes of MED subunits is not lethal in plants, but in turn leads to distinctive phenotypes that serve as valuable systems for characterizing the selected MED subunit and its involvement in plant-specific biological processes [33]. Our results provide a comprehensive portrait of the modulation of MED subunits in *N. tabacum*, *N. benthamiana* and hop infected with different viroid species. Nevertheless, the individual functional role of MED subunits in viroid infection remains to be clarified. To this end, we are currently working on a genome-editing-based functional characterization of selected viroid-responsive MED subunits and their interacting proteins in the model plant *N. benthamiana*. We hope that the results of these studies will provide informative insights into the role of MED subunits in viroid pathogenesis and the associated asymptomatic/symptomatic role in the plants, and open up a new avenue of managing viroid infections in commercially important plants.

## 3. Materials and Methods

In this study, hop (*Humulus lupulus* L.) cv. ‘Celeia’, *Nicotiana benthamiana* and tobacco (*N. tabacum*) plants were used as the main experimental hosts, and our previously constructed infectious cDNA dimeric constructs of AFCVd, CBCVd, PSTVd, HSVd and HLVd driven by the CaMV 35S promoter (Figure 1) [47] mobilized into an *Agrobacterium* strain (LBA 4404) was used for artificial viroid infection experiments. These infectious cDNA dimeric constructs (except HLVd) cause high plant mortality and morphological disorders in their host plants [31,47]. The wild-type (wt) *N. benthamiana* plants were grown from seeds until at the four-leaf stage and then mock or viroid (AFCVd, CBCVd and PSTVd)-inoculated (three leaves per plant) onto carborundum-wounded leaves. The systematically viroid-infected and mock-inoculated plants were grown under greenhouse conditions at 25 °C with supplementary illumination to maintain a 16 h photoperiod. Since tobacco is considered a non-host for PSTVd [48], the biolistic inoculation was only performed with cDNA dimeric constructs of AFCVd and CBCVd on fully expanded leaves of three-week-old tobacco plants (three leaves per plant), as described earlier [47]. Each tobacco plant was biolistically inoculated five times with approximately 50 ng cDNA per viroid species and was grown under the above-mentioned conditions for four to five weeks. The clonally propagated, three-month-old virus and viroid-free hop plants grown in a pot (10–14 cm height with at least three shoots) were biolistically inoculated five times with 250 ng cDNA of viroid (HLVd, CBCVd, HSVd) immobilized on microcarrier gold particles (1μm) following the previously described protocol [49]. The biolistic and mock-inoculated hop plants were covered with plastic bags to avoid the drying of the shot-wound leaf area and transferred to growing chamber conditions at 25 °C and 16 h illumination (90 μmol m^−2^s^−1^PAR).

Total RNA was extracted from 100 mg of mock-inoculated and systemically viroid-infected symptomatic (HSVd and CBCVd) and asymptomatic (HLVd) hop leaves (412 dpi), moderately symptomatic *N. benthamiana* (21 dpi) and asymptomatic tobacco (30 dpi) leaves using the Spectrum™ Plant Total RNA Kit (Sigma-Aldrich, St. Louis, MO, USA) followed by removal of the DNA contamination with the DNA-free^TM^ DNA Removal Kit (Ambion, Carlsbad, CA, USA) according to the manufacturers’ instructions. The presence of viroid in the leaf samples was confirmed by a combination of reverse transcription PCR (RT-PCR) and strand-specific real-time RT-qPCR (ssRT-qPCR) to determine the relative levels of (+) and (−) vd-sRNAs in viroid-infected plants.

To investigate the impact of viroid infection, 19 candidate MED subunits were selected based on their well-characterized functions in plants [32]. The MED proteins in the genomes of hop, tobacco and *N. benthamiana* were identified by National Center for Biotechnology Information (NBCI) BLAST_P_ (e-value cutoff of 1e^−5^) homology searches against the MED subunit protein sequences of *Arabidopsis*. The presence of the most prevalent highly conserved regions among hits and/or the presence of MED-specific Pfam domains was used as a screening criterion to identify MED subunits in hop, tobacco and *N. benthamiana* as described earlier [50]. The retrieved homologous sequence of the MED subunits was further confirmed by a hidden Markov model (HMM) method using the individual MED subunit domain. High-quality cDNAs were synthesized from 1 µg DNase-treated total RNA of the hop, *N. benthamiana* and *N. tabacum* with oligo (dT) primers using SuperScript IV Reverse Transcriptase (Invitrogen, Carlsbad, CA, USA).

The quantitative reverse transcription PCR (qRT-PCR) was performed with candidate MED subunit-specific primers (Table S1) in a CFX Connect™ Real-Time PCR Detection System (Bio-Rad, Hercules, CA, USA) using the TopBio SYBR master mix (TopBio, Prague, Czech Republic) with 3 μL of template cDNA (10-fold diluted). The relative transcriptional changes in gene expression levels (fold change) were calculated by the comparative Ct (2^−ΔΔCt^) method [51] using *DRH1* (DEAD-box ATPase-RNA-helicase) [49] as the reference gene. Three biological replicates were used for each sample and the experiment was repeated at least three times to confirm the reliability of the data.

## Supplementary Materials

Supplementary materials can be found at https://www.mdpi.com/1422-0067/21/7/2498/s1.
